# Lipid Conjugation of a Photoprotective Meadowfoam (*Limnanthes alba*) Glucolimnanthin Derivative Reduces Cytotoxicity, Attenuates UV-Induced DNA Damage and Activates DNA Repair in Human Keratinocytes Following UV Radiation

**DOI:** 10.3390/biom16081151

**Published:** 2026-08-07

**Authors:** Evan L. Carpenter, Wenbin Wu, Ewa Podgórska, Vajravathi Lakkim, Saiashish G. Singh, Andrzej T. Slominski, Gitali Ganguli-Indra, Jan F. Stevens, Arup K. Indra

**Affiliations:** 1 Department of Pharmaceutical Sciences, Oregon State University, Corvallis, OR 97331, USA; evan.carpen@berkeley.edu (E.L.C.); wbwu318@hotmail.com (W.W.); vajralakkim@gmail.com (V.L.); sgsingh43@gmail.com (S.G.S.); gitali.indra@oregonstate.edu (G.G.-I.); fred.stevens@oregonstate.edu (J.F.S.); 2 Linus Pauling Institute, Oregon State University, Corvallis, OR 97331, USA; 3 Department of Dermatology, University of Alabama at Birmingham, Birmingham, AL 35233, USA; epodgorska@uabmc.edu (E.P.); aslominski@uabmc.edu (A.T.S.); 4 Birmingham Veteran’s Affairs Medical Center, Birmingham, AL 35233, USA; 5 Molecular and Cellular Biology Program, Oregon State University, Corvallis, OR 97331, USA; 6 Knight Cancer Institute, Oregon Health and Science University, Portland, OR 97239, USA; 7 Department of Dermatology, Oregon Health and Science University, Portland, OR 97239, USA

**Keywords:** natural product, ultraviolet B (UVB)-induced damage, UVB-absorption, photoprotection, DNA damage response (DDR), skin

## Abstract

Ultraviolet B (UVB) radiation is a primary cause of DNA damage in the skin, which is often a precursor to skin cancer. Natural products represent a rich source of compounds with unexplored photoprotective properties. Our previous work identified 3-methoxybenzyl isothiocyanate (MBITC), a meadowfoam derivative, as a promising UVB-absorptive agent that reduces DNA damage and cell proliferation; however, its clinical use is limited by dose-dependent cytotoxicity. To address this, we synthesized a novel amide lipid conjugate of MBITC, N-(3-methoxybenzyl)eicos-5-enamide (MBA), and evaluated its photoprotective efficacy and mechanism of action. Our findings demonstrate that MBA exhibited remarkably reduced cytotoxicity compared to its parent compound, while effectively retaining its photoprotective properties. In human primary keratinocyte cultures, MBA significantly reduced UVB-induced DNA damage, as evidenced by a decrease in cyclobutane pyrimidine dimers (*p* < 0.05) and γ-H2A.X (*p* < 0.05). Furthermore, MBA increased the expression of DNA damage response (DDR) proteins and DNA damage-binding protein 1 (DDB1) (*p* < 0.05) and the activation of Ataxia Telangiectasia and Rad3-related protein (ATR) (*p* < 0.05), a master regulator of DDR and repair pathways. These findings suggest that MBA acts through direct UVB absorption and/or the engagement of DDR pathways following irradiation, highlighting its potential use as a novel photoprotective compound.

## 1. Introduction

Chronic overexposure of the skin to solar ultraviolet radiation (UVR) is a common dermatological concern that, without proper mitigation, causes cumulative damage that leads to premature photoaging and increased risk of cancer. Accordingly, skin cancers are the most frequently diagnosed form of cancer, and the burden of melanoma—the deadliest skin cancer—is expected to increase globally over the next couple of decades to 2040 [1]. UVR is known to be the primary initiator of melanomagenesis in at least three-quarters of cases, where key driver mutations in melanocyte protooncogenes, such as BRAF and NRAS, arise and initiate tumorigenesis [2,3]. Therefore, photoprotection of the skin is essential for combating the deleterious aspects of UVR.

Solar UVR is categorized into three wavelength ranges: UVA (315–400 nm), UVB (280–315 nm), and UVC (100–280 nm) [4]. The Earth’s ozone layer absorbs UVC wavelengths and UVB wavelengths shorter than 290 nm, preventing them from passing through the atmosphere and reaching the surface. As a result, only longer UVB (290–315 nm) and UVA wavelengths are able to penetrate the atmosphere and reach the Earth’s surface to irradiate our skin [5,6]. Both UVA and UVB wavelengths are damaging to the skin because they induce DNA damage that leads to cancer risk and premature skin aging. However, UVB is the more directly damaging of these two wavelengths because it predominantly acts through a chromophore-based mechanism that includes DNA and is primarily responsible for UV-induced sunburns and erythema [6,7]. One molecular mechanism by which UVB injures the skin is the direct induction of DNA damage in cells, resulting in mutagenic cyclobutane pyrimidine dimers (CPDs) and 6,4-photoproducts [8]. It also induces an immunosuppressive environment and production of some proinflammatory cytokines, which all contribute to a skin-carcinogenic environment [9]. For this reason, prospective photoprotective compounds ought to be examined for their capability to protect against UVB wavelengths.

Natural products offer a rich source of organic photoprotective phytochemicals that exhibit UV-absorbing or antioxidant properties, including polyphenols, flavonoids, pigments, and polysaccharides [10,11]. For example, the plant-derived pigment fucoxanthin has demonstrated photoprotective effects in retinal cells through its antioxidant properties, which mitigate UVB-induced reactive oxygen species and regulate MAPK, NF-κB, and Wnt signaling pathways [12,13]. The glucosinolate derivative sulforaphane exhibits photoprotective properties by activating NRF2, the key transcriptional regulator of cellular antioxidant responses, which leads to the upregulation of the glutathione and thioredoxin antioxidant systems [14,15]. UVB-absorptive properties have also been demonstrated, as seen with the cyanobacteria-derived oxylipins saclipin A and B [16]. Plant-derived phytochemicals can also exhibit UVB-absorbing properties, as observed with the ethanolic extract of rambutan (*Nephelium lappaceum* L.), which contains polyphenols such as ellagic acid, corilagin, and geraniin [17].

Meadowfoam (*Limnanthes alba*, Limnanthaceae), native to southern Oregon and northern California, is cultivated in the Willamette Valley of Oregon for its seed oil, which is primarily used in the manufacture of cosmetics by virtue of the oil’s long-chain fatty acids containing a single double bond (C20:1 typically makes up >60% of the oil). The seedmark contains glucosinolates and phytoecdysteroids [18]. There is commercial interest in the degradation products of the major glucosinolate, glucolimnanthin, as an herbicide and pesticide [19]. Our previous work demonstrated that derivatives of the principal meadowfoam glucosinolate, glucolimnanthin, exhibit UV photoprotective and anti-aging properties in human skin equivalents [20]. These compounds, 3-methoxybenzyl isothiocyanate (MBITC) and 3-methoxyphenyl acetonitrile (MPACN), were found to reduce UVB-induced DNA damage, proliferation, and matrix metalloproteinase expression. However, we also demonstrated that MBITC exhibited significant cytotoxic effects at higher concentrations, which could have several causes. For example, electrophilic compounds, such as MBITC, can react with various biomolecules (e.g., proteins and DNA), causing accumulated damage that is mutagenic and cytotoxic [21]. Alternatively, UV-absorptive molecules, like photoprotective and free-radical-scavenging pigment eumelanin, can also have damaging effects following excitation as they transfer energy to DNA and induce CPDs [22]. The reactivity of electrophilic MBITC with biomolecules or UVB-induced photosensitization could be potential mechanisms by which MBITC exerts its cytotoxicity. Therefore, we sought to modify the compound to mitigate this deleterious side effect. Lipid–drug conjugates are a well-established method for reducing toxicity, enhancing bioavailability, and facilitating formulation into various delivery systems [23]. Therefore, we hypothesized that conjugating MBITC with meadowfoam fatty acids would reduce the cytotoxicity of the compound while retaining its UV photoprotective properties. We speculated this would alter the subcellular localization of the compound, allowing MBA to more readily associate with lipid membranes rather than reacting with cytosolic or nuclear proteins and DNA.

In the present study, we synthesized a novel lipid conjugate of MBITC, N-(3-methoxybenzyl)eicos-5-enamide (MBamide or MBA), using meadowfoam fatty acids and evaluated the compound’s cytotoxicity and photoprotective properties in vitro using human keratinocyte cultures. We found that the novel lipid conjugate exhibited reduced cytotoxicity against primary human epithelial cells while retaining the photoprotective properties of the parental compound by (1) absorbing UVB light, (2) reducing UV-induced DNA damage, and (3) activating DNA repair pathways. Altogether, the lipid conjugation of a UVB-absorbing compound yielded an improved preliminary safety profile with a unique mechanism of action for photoprotection.

## 2. Materials and Methods

### 2.1. Synthesis of N-(3-Methoxybenzyl)eicos-5-enamide

Meadowfoam seed oil includes triglycerides whose fatty acids are primarily made up of eicos-5Z-enoic acid (C20:1; 61%), docos-5Z-enoic acid (C22:1; 16%), and do-cosa-5Z,13Z-dienoic acid (C22:2; 18%). Meadowfoam seed oil (1 mL) was transferred to a 50 mL round-bottom flask, and 5 mL of KOH (280 mg) solution in ethanol was added. The mixture was stirred for 3 h at 70 °C. After cooling, 3 mL of aqueous 2 N HCl and 12 mL of water were added, and the mixture was extracted three times with 20 mL of ethyl acetate. The organic layers were collected, dried over anhydrous Na_2_SO_4_, filtered, and taken to dryness by rotary evaporation. An aliquot of the hydrolyzed oil (150 µL) and 60 µL of MBITC (Oakwood Products, West Columbia, SC, USA) were transferred to a 2 mL glass vial, and the contents were vortexed for 20 s. The vial was capped, and the contents were heated for 6 h at 160 °C. After cooling, the mixture was diluted with 50 mL ethyl acetate and washed with saturated aqueous NaHCO_3_ (3 × 50 mL) and water (2 × 50 mL). The organic layer was dried over anhydrous Na_2_SO_4_, filtered, and taken to dryness by rotary evaporation, and the residue was reconstituted in 1 mL of ethyl acetate. The product was purified by open-column silica gel chromatography using hexane-ethyl acetate (5:1, 300 mL; 3:1, 400 mL; 1:1, 300 mL) and ethyl acetate (300 mL) as solvents. Fractions were collected and monitored by silica gel TLC developed in dichloromethane. The amide-containing fractions were combined and evaporated, and the product was analyzed by high-resolution mass spectrometry on a Sciex TripleTOF^®^ 5600 instrument (Sciex, Toronto, ON, Canada) operated in positive ion electrospray mode. The major product was consistent with the molecular formula (C_28_H_47_NO_2_, Δ = −3.4 ppm) and with the production of a methoxybenzyl fragment ion observed at *m*/*z* 121 (collision energy 40 eV). UV spectra of the isothiocyanate and the amide conjugate were obtained by HPLC with photo-diode array detection, as described in Stevens et al. 2009 [24]. The 1H NMR spectrum of the amide conjugate, N-(3-methoxybenzyl)eicos-5-enamide (MBA), was recorded in methanol-d4 (700 MHz) using a Bruker Ultrashield Plus 700 MHz spectrometer (Bruker Corporation, Billerica, MA, USA).

### 2.2. UVB Absorption Assay

MBITC or MBA was diluted in ethanol, added to separate wells of a 24-well plate (Corning, Corning, NY, USA), and irradiated with UVB light using a UV Transilluminator 2000 (Bio-Rad Laboratories, Hercules, CA, USA). A Daavlin X-96 irradiance meter (Daavlin, Bryan, OH, USA) measured energy transmittance through the dish.

### 2.3. Cell Culture

Immortalized keratinocyte HaCaT cells (ATCC, Manassas, VA, USA) were cultured in DMEM supplemented with 5% fetal bovine serum and 1% Antibiotic–Antimycotic (ThermoFisher Scientific, Waltham, MA, USA). HaCaT cells were tested for mycoplasma contamination using the Pro PCR Detection & Elimination Bundle kit (Applied Biological Materials, Inc., Richmond, BC, Canada). Human primary epidermal keratinocytes (HPEKs) (ATCC: PCS-200-011) were cultured in EpiLife medium (ThermoFisher Scientific) supplemented with Human Keratinocyte Growth Supplement (ThermoFisher Scientific). HPEKs were characterized and tested for mycoplasma by ATCC and were subcultured for fewer than ten passages. Cell lines were incubated in a humidified environment at 37 °C with 5% CO_2_.

### 2.4. Dose–Response and Time Kinetics Assays

For viability studies, HaCaT cells were seeded at 5000 cells/well in clear 96-well plates (Corning, Corning, NY, USA). After 24 h, cells were treated with MBITC or MBA and incubated with the compounds for 48 h. Viability was then determined using the MTS assay using the CellTiter 96 Aqueous One Solution Cell Proliferation Assay Kit (Promega, Madison, WI, USA). Absorption was measured using a Cytation 5.0 Imaging Reader (Agilent, Santa Clara, CA, USA). HPEKs were seeded 5000 cells/well into white, opaque-bottom 96-well plates (Greiner Bio-One, Monroe, NC, USA) and incubated for 48 h. HPEKs were treated with ethanol vehicle control or a 5-point half-log serial dilution of MBA starting at 50 μM. After 48 h, CellTiter-Glo (Promega) viability assays were performed, and luminescence was read using a Synergy4 (Biotek, Winooski, VT, USA) plate reader.

For UV protection dose–response assays, HaCaT cells were seeded 5000 cells/well into clear 96-well plates (Corning). After 24 h, cells were treated with MBITC or MBA and incubated for 12 h. Cells were washed in PBS and irradiated with UVB using a UV Transilluminator 2000. After irradiation, fresh media was added, cells incubated for 36 h, and viability was determined by MTS assay. HPEKs were seeded at 5000 cells/well into black, opaque-bottom 96-well plates (Greiner Bio-One) and incubated for 48 h. Afterward, cells were treated with a vehicle control, MBITC, or MBA and incubated for 12 h. Cells in PBS supplemented with compounds were irradiated using UVB TL 20W/12 RS M3 lights (Philips, Amsterdam, Netherlands). The dose of UVB was quantified using an IL1400A NIST Traceable Photometer (International Light, Peabody, MA, USA). Following irradiation, the PBS was aspirated from the wells, and conditioned media was added. After 36 h, CYQUANT (ThermoFisher Scientific) assays were performed per the manufacturer’s instructions, and fluorescence was read using a Synergy4 plate reader. Cell counts were extrapolated from a standard curve of fluorescent signal activity vs. HPEK cell counts.

### 2.5. Immunocytochemistry

For CPDs, HPEKs were cultured to 80% confluence in 6-well plates containing gelatin-coated coverslips and treated with ethanol vehicle control or MBA. After 12 h, plates were irradiated with 5 mJ/cm^2^ UVB in PBS supplemented with EtOH or MBA and re-incubated with the original conditioned media for 24 h. For 8-oxo-dG and p-ATR staining, cells were treated with compounds for 16 h, irradiated with 5 mJ/cm^2^ UVB and incubated with the original conditioned media for 3 h. Cells were then fixed in 75% methanol/25% acetic acid, washed with 70% EtOH, and their DNA was denatured in 70 mM NaOH (in 70% EtOH). Wells were washed with PBS and denaturation was quenched with 100 mM Tris-HCl (in 70% EtOH). Cells were blocked with 10% normal goat serum in PBS-T and incubated overnight at 4 °C with either α-thymine dimer primary antibody (1:1000, Kamiya Biomedical, Matsumoto, Nagano, Japan, catalogue: MC-062), α-8-hydroxy-2′- deoxyguanosine primary antibody (1:250, R&D Systems, Inc., Minneapolis, MN, USA, catalogue: 4354-MC-050), or α-p-ATR (1:250, Cell Signaling, Danvers, MA, USA, catalogue: 2853). Samples were then incubated with Cy3 goat α-mouse (1:50, Jackson Immunolabs, West Grove, PA, USA, catalogue: 115-165-003) or goat α-rabbit secondary antibody (Jackson Immunolabs, catalogue: 111-225-144). Cells were counterstained with 0.2 ng/mL DAPI and dehydrated through an ethanol gradient, and coverslips were mounted to slides using dibutyl phthalate in xylene (VWR, Radnor, PA, USA).

### 2.6. Microscope Image Acquisition and Quantification

Fluorescent microscopy used a Zeiss AXIO Imager.Z1 (Carl Zeiss, Oberkochen, Germany) with 20X magnification and 0.8 numerical aperture. For each independent experiment, treatments were performed with technical duplicates, and 10 fields were imaged per replicate. Counts of cells were performed by two investigators blinded to the experimental groups of the samples. Corrected total cellular fluorescence (CTCF) was quantified from unprocessed images using the ImageJ software (version 1.54f) package as described previously, except only nuclear CTCF was quantified and nuclei were selected for ROIs using manual thresholding for each field [25]. Cells on the edges of each field were excluded for all quantifications.

### 2.7. Immunoblotting

HPEKs were cultured to 80% confluence in 6-well plates and treated with ethanol vehicle control or MBA. After 12 h, cells were irradiated with 5 mJ/cm^2^ UVB, and 12 or 24 h after treatment, cells were harvested by scraping into cold PBS, centrifuged, pellet resuspended in denaturing SDS lysis buffer, boiled, and sonicated. Samples were normalized to protein content as determined by BCA Assay (ThermoFisher Scientific). SDS-PAGE was then performed using Novex^TM^ 4–20%, Tris-Glycine Plus WedgeWell^TM^ gels (ThermoFisher Scientific) and transferred to a 0.45 μm PVDF membrane (Merck Millipore Ltd., Cork, Ireland) or 0.2 μm nitrocellulose membrane (Bio-Rad Laboratories) for blots probed for γ-H2A.X. Blots were blocked for 1 h in 5% nonfat dry milk in TBS-T and then incubated with α-phospho-ATR (S428) (1:500, ThermoFisher Scientific, catalogue: PA5-121295), α-DDB1 (1:1000, Novus Biologicals, LLC., Centennial, CO, USA, catalogue: NBP1-33061), XPC (1:1000, Cell Signaling Technology, Danvers, MA, USA, catalogue: 14768S), γ-H2A.X (Cell Signaling Technology, catalogue: 9718S), or Actin (1:10,000, Bethyl Laboratories, Inc., Montgomery, TX, USA, catalogue: A300-491A) diluted into 5% milk in TBS-T overnight at 4 °C. The next day, blots were incubated with HRP-conjugate donkey anti-rabbit secondary antibody (1:5000, Cytiva, Marlborough, MA, USA, catalogue: NA934) for 1 h. The immunoblots were developed using WesternBright^TM^ Sirius chemiluminescent reagent (Advansta, Inc., San Jose, CA, USA) and imaged with a ChemiDoc^TM^ MP Imaging System (Bio-Rad Laboratories).

### 2.8. Software and Statistics

The imaging software used for fluorescence microscopy was AxioVision 4.8 (Carl Zeiss), and fluorescence images were processed using Photoshop (Adobe, San Jose, CA, USA) with “Brightness/Contrast” and “Levels” image adjustments. CTCF was quantified using ImageJ 1.54f (National Institutes of Health, Bethesda, MD, USA). Densitometry of immunoblots was performed using Image Lab Software (Bio-Rad Laboratories). Statistics were performed with Prism 10 (GraphPad Software, San Diego, CA, USA). A one-way ANOVA was performed for experiments comparing MBA and MBITC so that both could be factored into the analysis of the difference from the vehicle control. When fold changes were calculated for experiments in which only MBA was used, a one-sample *t*-test was performed.

## 3. Results

**Figure 1:**
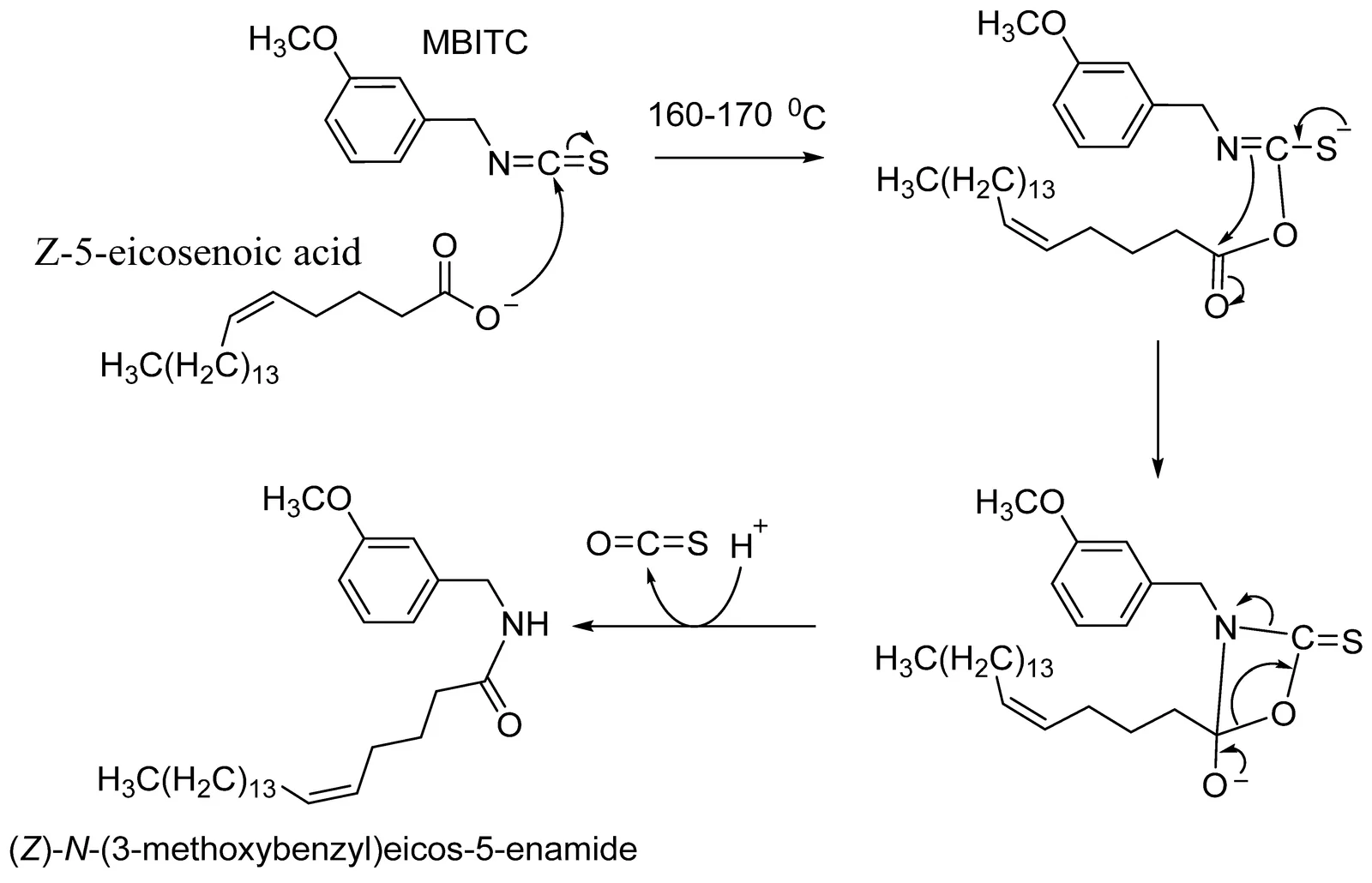
Schematic representation of the synthesis of N-(3-methoxybenzyl)eicos-5-enamide (MBamide or MBA) from MBITC and the meadowfoam fatty acid, Z-5-eicosenoic acid.

**Figure 2:**
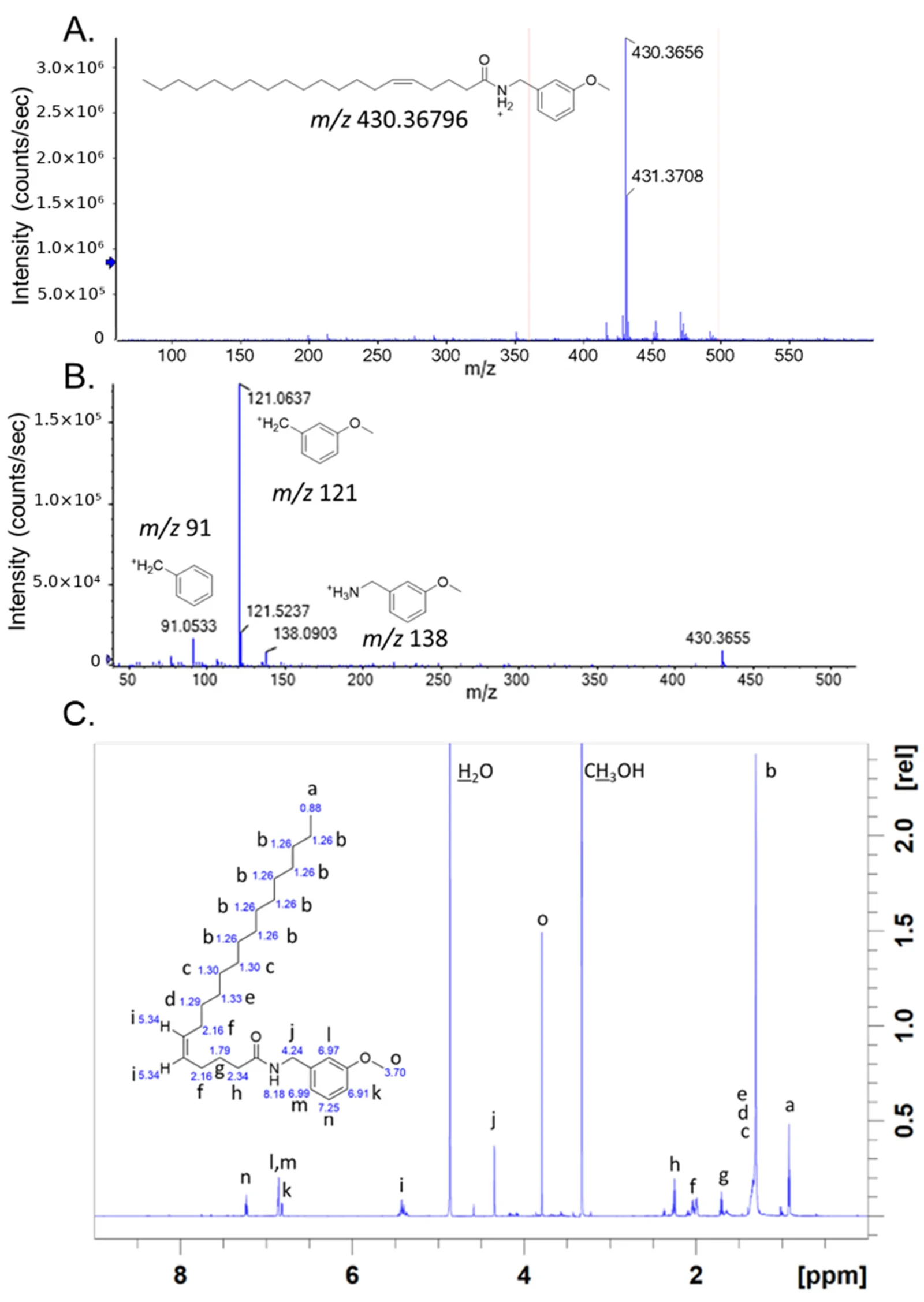
Characterizing the structure of the novel lipid-conjugated compound. (**A**) Time-of-Flight (ToF) mass spectrum of N-(3-methoxybenzyl)eicos-5-enamide (MBA), prepared from 3-methoxybenzyl isothiocyanate (MBITC) and meadowfoam-derived fatty acids (Observed: *m*/*z* 430.3656; calculated for [C28H47NO2]+: *m*/*z* 430.36796; mass error −5.4 ppm). (**B**) Q-ToF MS/MS spectrum of the molecular ion from panel A showing the expected mass fragments with *m*/*z* 138, 121, and 91. (**C**) 1H NMR Spectrum of N-(3-methoxybenzyl)eicos-5-enamide (MBA) in methanol-d4 (700 MHz). The structure of MBA shows predicted chemical shifts (ppm) and annotated protons.

Synthesis of the novel compound required the reaction of parental compound MBITC with meadowfoam fatty acids to form N-(3-methoxybenzyl)eicos-5-enamide (MBA) and related fatty acid-derived amides. To do so, meadowfoam oil was saponified, and the resulting mixture of meadowfoam fatty acids (MFAs) was isolated. Using established procedures reported in the literature, MBITC was mixed with MFAs in equimolar amounts, and the mixture was heated at 160 °C for 6 h with magnetic stirring (Figure 1). LC-MS/MS was used to monitor the formation of MBITC-MFA amide conjugates. After optimizing the yield under various conditions, we produced conjugates on a larger scale (0.1–1 g). The products were purified by column chromatography to remove residual MBITC and MFAs. The structure of MBA was confirmed using Time-of-Flight (ToF) mass spectrometry and proton nuclear magnetic resonance (1H NMR) (Figure 2).

**Figure 3:**
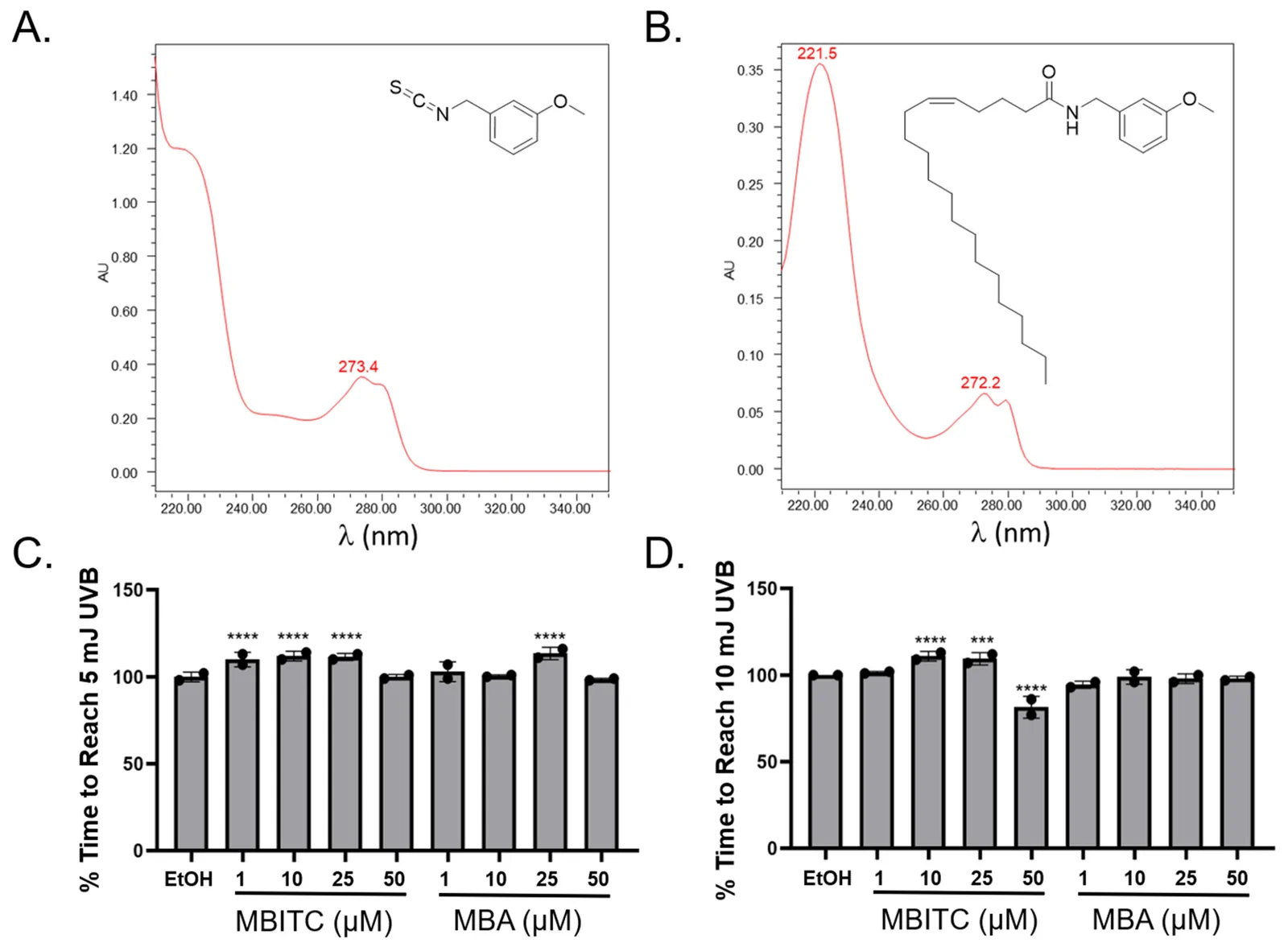
Novel lipid-conjugated compound retains significant UV absorbency. UV Spectra of (**A**) 3-methoxybenzyl isothiocyanate (MBITC) and (**B**) N-(3-methoxybenzyl)eicos-5-enamide (MBA), showing that the benzyl chromophore remains intact after acyl conjugation of MBITC (max 273 nm). UVB absorption assay for MBITC and MBA, measuring the relative time required to reach 5 mJ/cm^2^ (**C**) or 10 mJ/cm^2^ (**D**). Data represent mean ± SD from two independent experiments (**C**,**D**). Dots on bar graphs indicate experimental replicates. Significance determined by one-way ANOVA. *** = *p* < 0.001, **** = *p* < 0.0001.

We then sought to determine if MBA retained UV-absorptive capability following lipid conjugation by measuring its UV absorption spectra. MBA exhibited an absorption peak around 280 nm in the UVB wavelengths (280–315 nm) besides another peak around 272 nm, which is similar to parental MBITC (Figure 3A,B). We further confirmed this observation by performing a dose–response UVB absorption assay, in which we observed that the light absorbed by MBITC was sufficient to increase the time required to reach a 5 mJ/cm^2^ dose at every tested concentration, except at 50 μM. On the other hand, MBA only showed similar activity at a single concentration of 25 μM (Figure 3C). A similar trend was also observed in the time required to reach 10 mJ/cm^2^ (Figure 3D). These data indicate the preserved UVB absorption capacity of MBA and suggest that lipid conjugation might attenuate the compound’s absorptive capacity.

**Figure 4:**
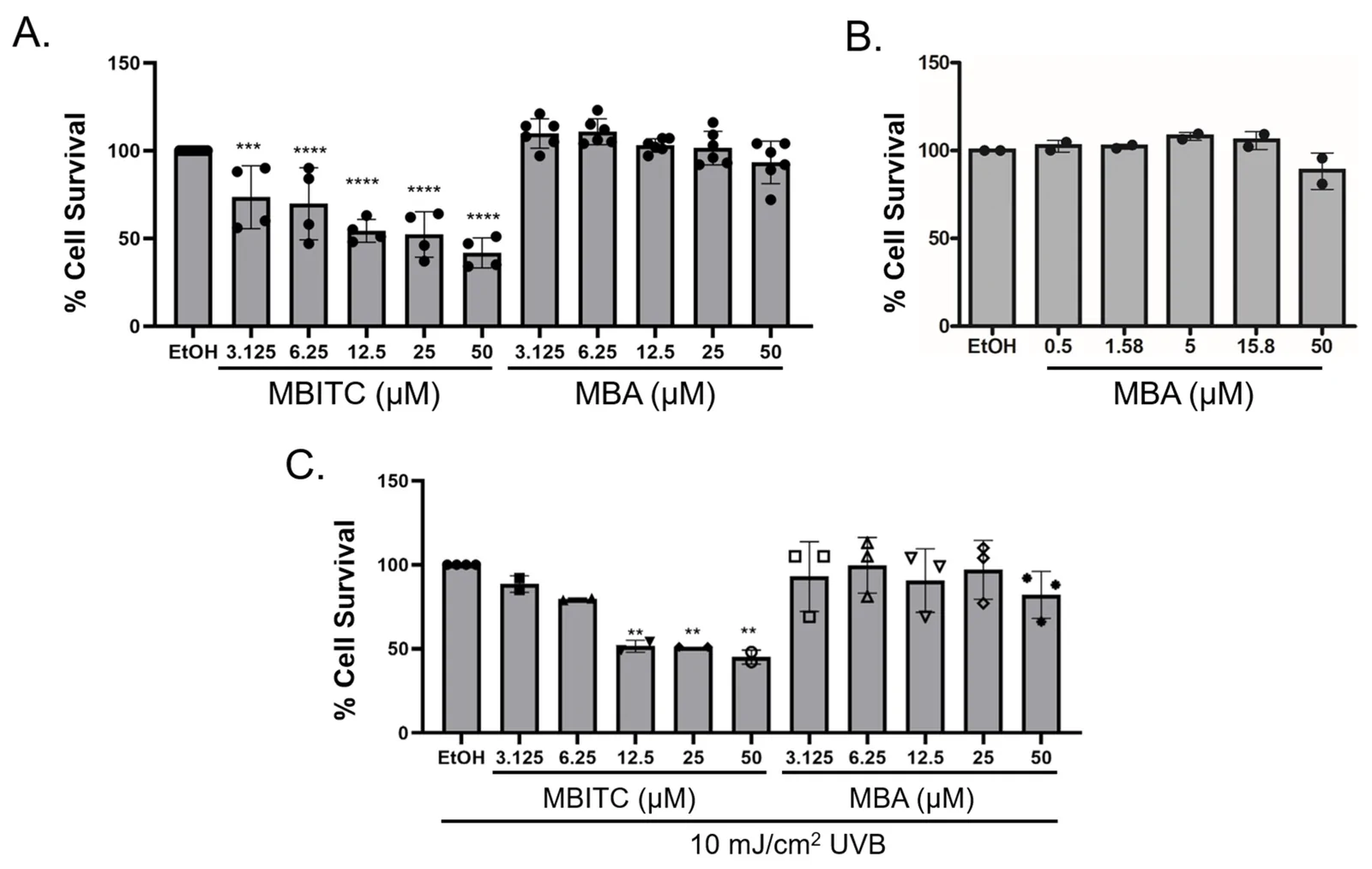
MBA exhibits no significant cytotoxicity in human keratinocyte cultures. (**A**) Dose–response MTS assay for survival of HaCaT cells incubated with MBITC or MBA for 48 h. Four and six independent experiments with technical triplicates were performed for MBITC and MBA, respectively. (**B**) Dose–response Cell Titer-Glo assay for survival of human primary epidermal keratinocytes treated with MBA for 48 h. Two independent experiments were performed with technical triplicates. (**C**) HaCaT cells were treated with EtOH, MBITC, or MBA for 12 h, then irradiated with 10 mJ/cm^2^ UVB, and then an MTS survival assay was performed after 36 h. Two and three independent experiments were performed with technical triplicates for MBITC and MBA, respectively. Data represent mean ± SD. Dots on bar graphs indicate experimental replicates. Assorted shapes are used to indicate different doses of MBITC and MBA in the presence of UVB. Significance determined by one-way ANOVA. ** = *p* < 0.01, *** = *p* < 0.001, **** = *p* < 0.0001.

To examine the effect of lipid conjugation on the cytotoxicity of the novel compound, we performed dose–response viability assays using MBA and MBITC in the absence of UVB irradiation. We have previously observed that MBITC exhibited cytotoxic effects in human primary epidermal keratinocytes (HPEKs) under these conditions. In immortalized human keratinocytes (HaCaT cells), we observed that MBITC exhibited significant cytotoxicity at all the tested doses, whereas MBA had little to no effect (Figure 4A). Similarly, MBA was also not cytotoxic at any of the tested doses in HPEKs (Figure 4B). Overall, these data illustrate that lipid conjugation of MBA abrogates the deleterious cytotoxic effect observed with the parental compound MBITC. We then tested the ability of both compounds to recover HaCaT cells from a UVB-induced temporary halt of proliferation. Similar to our previous findings, MBITC did not recover the pause of cell proliferation following irradiation with 10 mJ/cm^2^ UVB and exhibited further detrimental cytotoxic effects in the presence of UVB (Figure 4C). Interestingly, MBA did not show any significant cytotoxic effects compared to the parental compound at the tested concentrations. We further examined the capability of MBA to recover UVB-induced pause of proliferation in primary human keratinocytes (HPEKs), where the compound exhibited similar effects as observed in HaCaT cells (Figure S1A). Increasing the incubation time to four days did not reveal significant protective effects of MBA against UVB-induced inhibition of cell proliferation under these conditions (Figure S1B).

**Figure 5:**
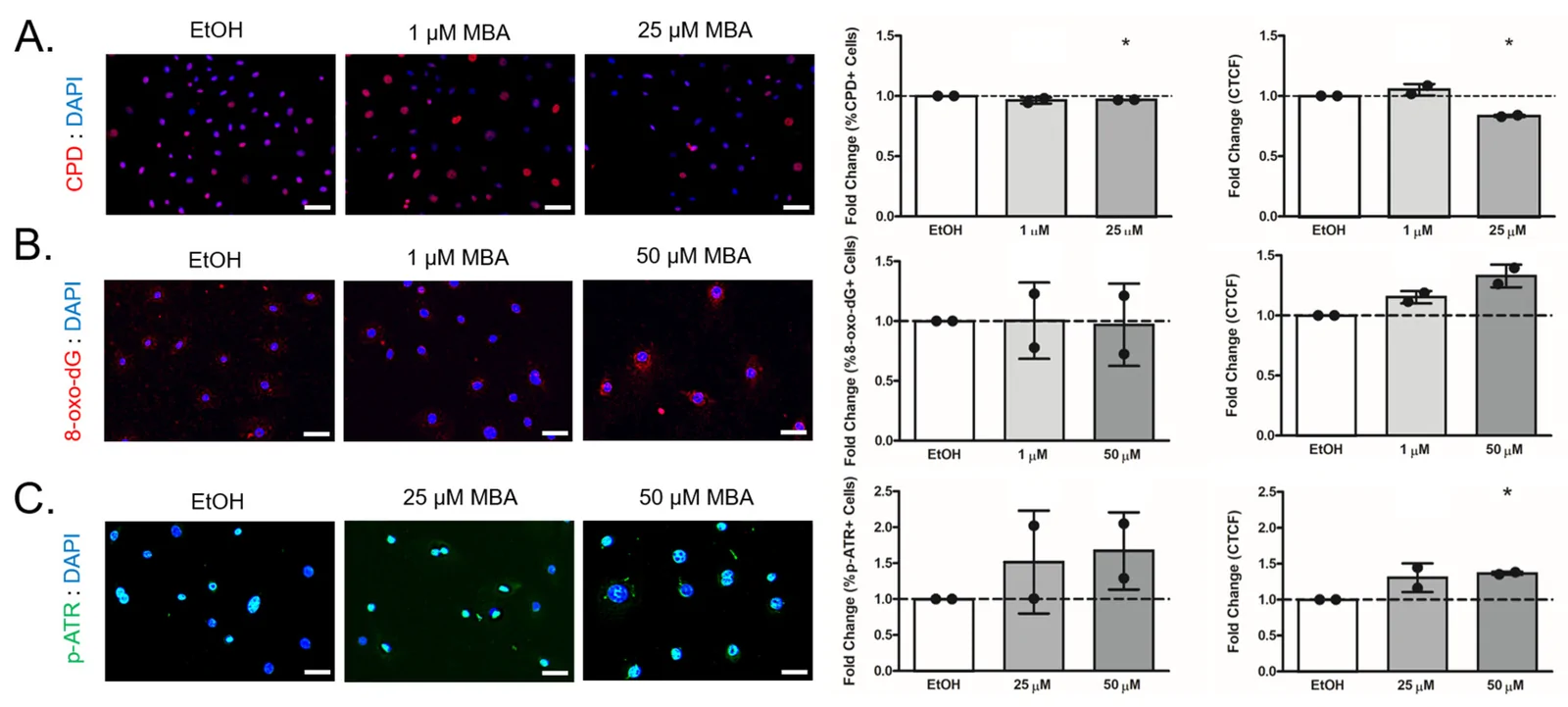
MBA significantly ameliorates UVB-induced DNA damage and increases ATR activation. Immunocytochemistry for (**A**) CPDs (red), (**B**) 8-oxo-dG (red), and (**C**) p-ATR (green) in human primary keratinocytes treated with EtOH or indicated doses of MBA for 12 h (**A**) or 16 h (**B**,**C**), followed by irradiation with 5 mJ/cm^2^ UVB and then fixation after 24 h (**A**) or 3 h (**B**,**C**). Scale bars indicate 50 μm. Quantification for each immunocytochemistry staining was performed to determine the percentage of positive cells and nuclear fluorescence intensity using corrected total cellular fluorescence (CTCF). Data represent mean ± SD from two independent experiments performed with technical duplicates. Dots on bar graphs indicate experimental replicates. Dotted lines indicate a fold change of 1. Significance was determined by a one-sample *t*-test for a difference from 1.0. * = *p* < 0.05.

Our previous study demonstrated that MBITC can prevent UV-induced DNA damage, such as cyclobutane pyrimidine dimers (CPDs) in UVB-irradiated human skin equivalents. Therefore, we were interested in determining MBA’s capability in this regard, while also examining an additional marker for DNA damage response (DDR) (e.g., cell cycle arrest, DNA repair, and apoptosis) by labeling for phosphorylated Ataxia Telangiectasia and Rad3-related protein (p-ATR) [26,27]. We found that 24 h following 5 mJ/cm^2^ UVB irradiation, MBA treatment significantly reduced both the percentage of CPD+ cells and CPD nuclear fluorescence intensity at a 25 μM dose (Figure 5A). This aligned with our earlier observation of significant UVB absorption at this dose, indicating that MBA’s absorptive activity is sufficient to prevent UVB-induced DNA damage.

Interestingly, immunocytochemical staining for the DNA oxidative stress marker 8-oxo-2′-deoxyguanosine (8-oxo-dG) 3 h post-UVB showed no significant difference with MBA treatment (Figure 5B). There was a dose-dependent trend of increased 8-oxo-dG staining when signal intensity was quantified using CTCF analysis, suggesting that at doses higher than 50 μM, MBA could induce oxidative stress. However, it should also be noted that the percentage of 8-oxo-dG+ cells per field was relatively low (5–15%) compared to CPD+ (75–100%), indicating that overall DNA oxidative stress under these conditions was relatively low.

We next examined the activation of ataxia telangiectasia and Rad3-related protein (ATR)-dependent DNA repair by staining for its phosphorylated form p-ATR 3 h after UVB irradiation. We observed a clear trend of increased p-ATR with higher MBA doses, which was particularly evident in the significant increase in nuclear fluorescence intensity observed at a 50 μM dose (Figure 5C). This result suggests that the presence of MBA during UVB irradiation may improve subsequent DNA repair responses.

**Figure 6:**
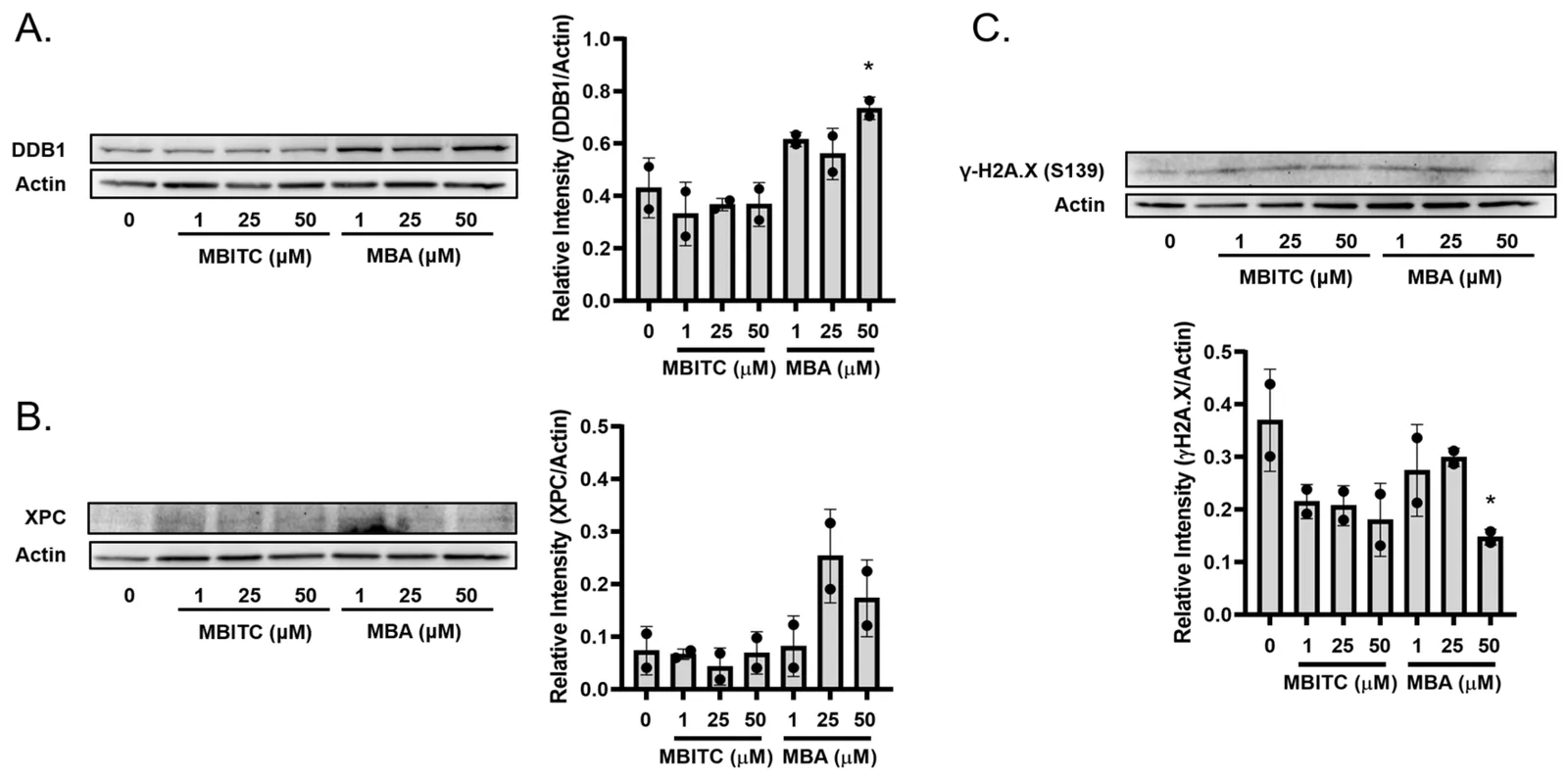
MBA treatment results in significantly higher expression of DDR-related protein DDB1 and reduced γ-H2A.X. Immunoblots and densitometric quantification for (**A**) DDB1, (**B**) XPC, or (**C**) γ-H2A.X (S139) in MBITC- or MBA-treated HPEK cell lysates harvested 12 h after irradiation with 5 mJ/cm^2^ UVB. Data represent mean ± SD from two independent experiments. Dots on bar graphs indicate experimental replicates. Significance determined by one-way ANOVA. * = *p* < 0.05. Original Blot images can be found in Supplementary Materials.

To further probe potential changes in DDR pathways during treatment with MBITC and MBA, we immunoblotted for DNA damage-binding protein 1 (DDB1) and xeroderma pigmentosum, complementation group C (XPC) 12 h following 5 mJ/cm^2^ UVB irradiation. We found significantly increased DDB1 expression at the highest dose of MBA treatment, whereas MBITC had no effect (Figure 6A). XPC showed a trend of increased expression with MBA treatment, which was not similarly observed with MBITC (Figure 6B). To examine general genomic stress, we also probed for the presence of γ-H2A.X, which is phosphorylated during several events, including DNA double-strand breaks, stalled/collapsed replication forks, and apoptosis [28]. We observed a significant decrease in γ-H2A.X following only MBA treatment at the highest dose (Figure 6C).

**Figure 7:**
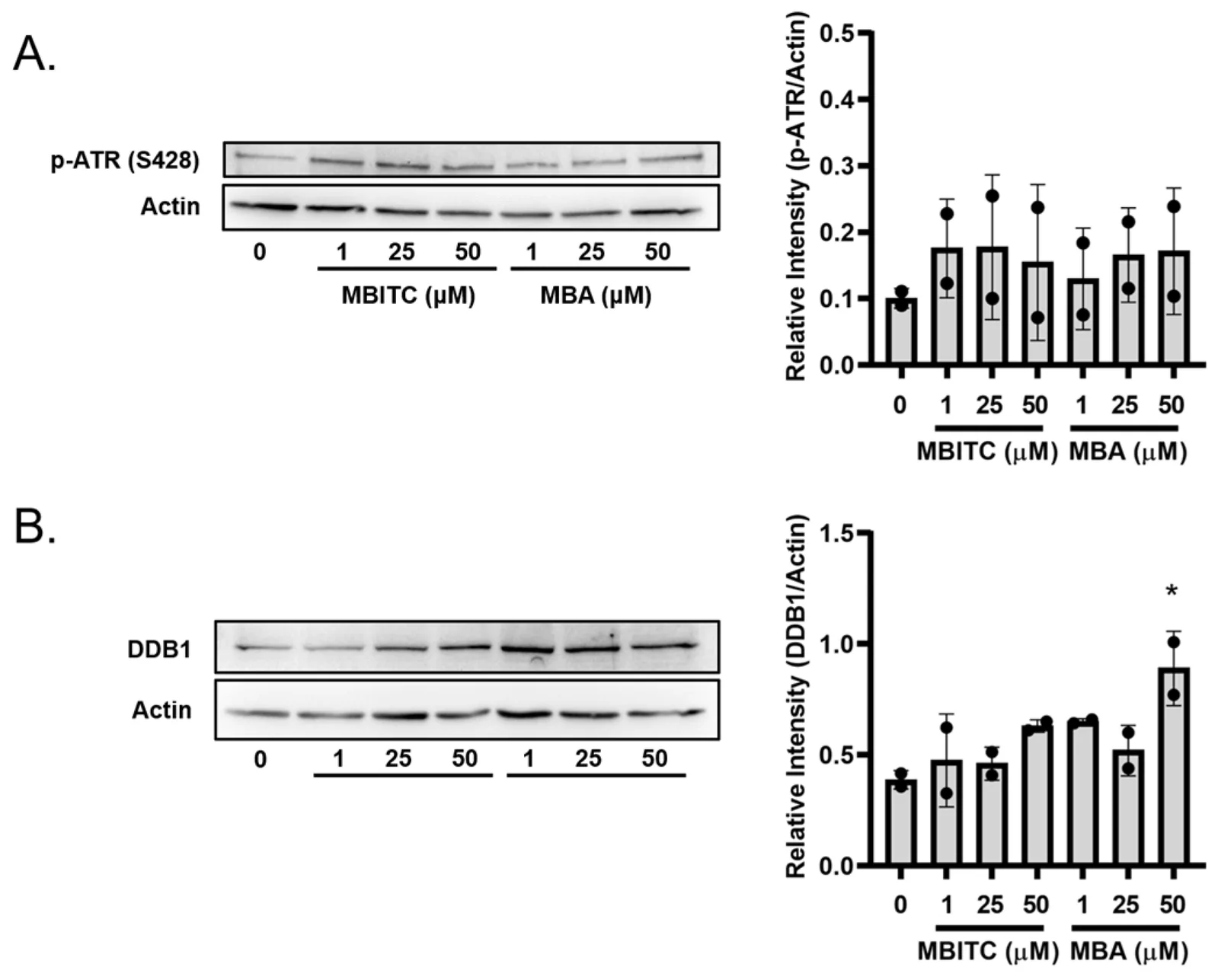
MBA treatment yields significantly higher DDB1 and a trend of increased ATR activation. Immunoblots and densitometric quantification for (**A**) phosphorylated ATR (S428) or (**B**) DDB1 in MBITC- or MBA-treated HPEK cell lysates harvested 24 h after irradiation with 5 mJ/cm^2^ UVB. Data represent mean ± SD, from two independent experiments. Dots on bar graphs indicate experimental replicates. Significance determined by one-way ANOVA. * = *p* < 0.05. Original Blot images can be found in Supplementary Materials.

Timepoints beyond 12 h were also tested to determine the prolonged effect of MBA on DDR pathways. To do so, we immunoblotted for p-ATR and DDB1 24 h post-UVB irradiation. We found a trend of increased p-ATR above the vehicle control at all tested doses of MBITC and MBA (Figure 7A) and a significant increase in the expression of DDB1 only at the highest dose of MBA (Figure 7B). These data show that MBA is likely to promote prolonged induction of DDR pathways. We also probed changes in the activation of p53 and found no difference between the compounds at any of the tested doses and the vehicle control.

## 4. Discussion

In this study, we synthesized a novel derivative of a photoprotective compound that improved its preliminary safety profile while retaining its photoprotective properties. MBA exhibited reduced cytotoxicity in both HaCaT and HPEK cultures, and we speculate that this is due to an altered intracellular distribution of the lipid-conjugated compound. By increasing its lipophilicity, MBA may more readily associate with lipid membranes, thereby reducing harmful interactions with nuclear DNA as well as nuclear and cytoplasmic proteins.

### 4.1. Mechanisms of Photoprotection

There are two potential mechanisms by which MBA may exert its photoprotective effects. The first is through direct absorption of UVB light by the compound itself, and the second is by engaging DDR pathways in the time following irradiation.

We demonstrated that MBA absorbs UVB wavelengths (280–315 nm), albeit at a reduced capacity as compared to MBITC. This diminished absorption is likely attributable to the loss of double bonds in the isothiocyanate side chain following lipid conjugation, a structural feature well-documented to contribute to UV absorption [29]. Despite its lower intrinsic UV absorbance, the photoprotective efficacy of MBA was confirmed via immunofluorescence staining for markers of UVB-induced DNA damage, showing reduced CPD formation without significantly affecting 8-oxo-dG levels. This distinct profile indicates that the compound likely operates through a UVB-absorptive mechanism rather than acting as a traditional antioxidant. Other lipid-conjugated photoprotective compounds have been developed with a similar mechanism of action. Rana et al. screened an assortment of compounds comprising a lipophilic alkyl chain conjugated to a thiourea-substituted aromatic ring and found that these structures could prevent UVA-induced DNA damage [30]. However, the compounds identified in that study exhibited more cytotoxicity than MBA, with IC50 values around 50 μM. At that same concentration, MBA did not show significant cytotoxic effects. Another study by Slabu et al. investigated a vegetable oil–UV filter conjugate that absorbed UVB wavelengths [31]. No bioassays were performed in that study, so the feasibility of the compounds from a cytotoxicity perspective was not determined.

We also observed that MBA treatment following UVB irradiation leads to altered DDR markers; we observed a dose-dependent increase in phosphorylated ATR, which serves as a critical molecular beacon, indicating that a cell’s DNA integrity has been compromised and its DNA damage response machinery has been engaged [26,27]. While it remains unclear whether MBA acts primarily through direct UVB absorption or DDR activation, comparing its activity to the parent compound, MBITC, provides key insights.

### 4.2. Relative Effects of MBA and MBITC on DNA Damage Response Pathways

As previously mentioned, UVB irradiation of the skin leads to the formation of DNA damage in the form of CPDs and 6,4-photoproducts [8]. These forms of DNA damage are recognized by the heterodimeric UV-damage DNA-binding protein (UV-DDB), formed by the association of DDB1 and DDB2. UV-DDB then recruits CUL4 (A or B) and ROC1 to assemble a functional E3 ubiquitin ligase complex [32]. This complex scans the genome for UV-induced DNA damage (including CPDs and 6-4 photoproducts), binds to the damaged DNA, and initiates nucleotide excision repair (NER) via ubiquitination of histone proteins and others, including XPC. Ubiquitinated XPC subsequently recruits additional NER machinery to the DNA damage lesion site, leading to nuclease excision of the damaged DNA strand and exposing a single-strand gap in the DNA [33,34]. This gap is coated by replication protein A (RPA), which is the triggering event for ATR recruitment and its activation by autophosphorylation [35]. Activated ATR then phosphorylates downstream targets, including Chk1, and the ATR-Chk1 pathway protects the genome against DNA damage and replication stress by regulating key cellular processes such as cell cycle progression [27,36]. In the context of DDR, the activation of ATR is a pro-survival indicator for the cell, which is crucial for the recruitment and assembly of the DNA repair complexes [37,38]. Further, activation initiates a signaling cascade that leads to cell cycle arrest, allowing time for DNA repair, and stabilizes replication forks to prevent genomic collapse [27,39,40].

Our results indicate that MBA, rather than MBITC, allows for significantly increased DDB1 expression and a trend of increased XPC expression up to 12 h—or in the case of DDB1, up to 24 h—after UVB irradiation. This suggests a relatively prolonged engagement of early DDR proteins that could allow for the increased repair of CPDs observed 24 h after irradiation, as well as the reduced γ-H2A.X seen at the 12 h timepoint. Generally, γH2A.X forms quickly, within minutes after UV irradiation, and is dependent on kinases such as ATM and JNK [41,42]. However, it has also been shown that the increased and persistent γH2A.X staining observed following UV irradiation serves as a pre-apoptotic indicator [43]. Therefore, MBA contributes towards clearing the residual γ-H2A.X that remains for hours after UVB exposure. Altogether, these observations indicate that MBA has two potential mechanisms by which it may afford photoprotection, whereas the parental compound MBITC likely acts solely through UVB absorption.

The precise molecular mechanism driving elevated DDB1 expression remains to be fully elucidated. While DDB2 is transcriptionally regulated by p53 with a binding site in its 5′-UTR [44,45], we detected no significant differences in p53 activation following MBA treatment, suggesting that DDB2 expression may not be similarly upregulated. However, alternative pathways may explain the reason for prolonged and elevated DDB1 expression. A recent study by Lim et al. found that DDB1 interacts with NBS1, which is a part of the MRE11-RAD50-NBS1 (MRN) complex [46]. Interestingly, the MRN complex is at least partially involved in the activation of ATR, which thereby links DDB1 expression and ATR activation during DDR [47]. Furthermore, DDB1 maintains critical, DDB2-independent functions, including transcription-coupled nucleotide excision repair (TC-NER) and participation in other CUL4A E3 ubiquitin–ligase complexes [48].

## 5. Conclusions

Overall, we demonstrate the generation of a novel photoprotective natural compound, MBA, via lipid conjugation of the parental compound MBITC, which reduces cytotoxicity while retaining photoprotective properties. MBA could exert its photoprotective effect in vitro through two mechanisms: as a UVB filter and/or by engaging DDR pathways. While MBITC and MBA absorb in the 280–290 nm range, ambient UVB reaching the skin at sea level is typically restricted to wavelengths above 290 nm. Therefore, future work is necessary to refine MBA’s chemical structure to enhance its absorbance across a broader range of UVB wavelengths while retaining its effects on DDR markers, with the ultimate goal of developing a new sunscreen compound. Furthermore, MBA and its derivatives could be tested in a more skin-like environment, such as reconstructed skin or bioprinted human skin, and in various formulations, including solid lipid nanoparticles.

## 6. Patents

Patent Pending. Submitted a non-provisional US Patent Application No. 19/015,263, entitled “Sunscreen Compounds and Formulations”, on 9 January 2025.

## Data Availability

The original contributions presented in this study are included in the article/Supplementary Material. Further inquiries can be directed to the corresponding author.

## Supplementary Materials

The following supporting information can be downloaded at: https://www.mdpi.com/article/10.3390/biom16081151/s1, Figure S1: MBA photoprotection assay in human primary epidermal keratinocytes (HPEKs). Original Blot images can be found in Supplementary Materials.

## Abbreviations

The following abbreviations are used in this manuscript:

UVR	Ultraviolet Radiation
MBITC	3-methoxybenzyl isothiocyanate
MPACN	3-methoxyphenyl acetonitrile
MBA	N-(3-methoxybenzyl)eicos-5-enamide
MFA	Meadowfoam fatty acid
ATR	Ataxia Telangiectasia and Rad3-related protein
DDB1	DNA damage-binding protein 1
XPC	Xeroderma pigmentosum, complementation group C
DDR	DNA damage response
CPD	Cyclobutane pyrimidine dimer
8-oxo-dG	8-oxo-2′-deoxyguanosine
HPEK	Human primary epidermal keratinocyte
CTCF	Corrected total cellular fluorescence
γ-H2A.X	Gamma-H2A histone family member X
