# Supplementary material for: Lipid Conjugation of a Photoprotective Meadowfoam (Limnanthes alba) Glucolimnanthin Derivative Reduces Cytotoxicity, Attenuates UV-Induced DNA Damage and Activates DNA Repair in Human Keratinocytes Following UV Radiation
Source: Biomolecules. 2026 Aug 7;16(8):1151. doi: 10.3390/biom16081151 (PMC13510429; doi:10.3390/biom16081151)
Supplement: Supplementary file 1 [file biomolecules-16-01151-s001.zip › biomolecules-4393290-supplementary.pdf]

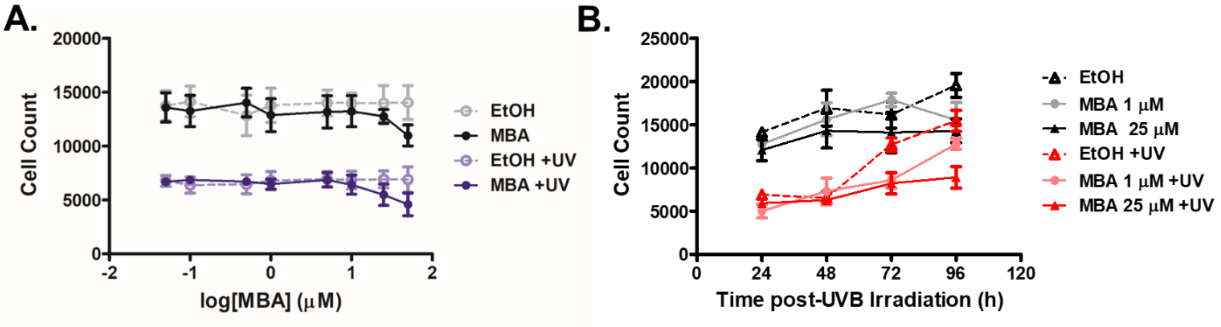


**Supplementary Figure S1.** MBA photoprotection assay in human primary epidermal keratinocytes (HPEKs). Cell proliferation assays for (A) dose-response of MBA after two days (48 hrs) of treatment or (B) time kinetics of proliferation of HPEKs treated with indicated doses of MBA for 12 h, in presence or absence of 10 mJ/cm2 of UVB irradiation. CyQuant cell proliferation assay was performed at indicated time points. Data represent mean ± SD from three independent experiments.
